# Capturing the response of *Clostridium acetobutylicum* to chemical stressors using a regulated genome-scale metabolic model

**DOI:** 10.1186/s13068-014-0144-4

**Published:** 2014-10-14

**Authors:** Satyakam Dash, Thomas J Mueller, Keerthi P Venkataramanan, Eleftherios T Papoutsakis, Costas D Maranas

**Affiliations:** Department of Chemical Engineering, The Pennsylvania State University, University Park, Pennsylvania USA; Delaware Biotechnology Institute, 15 Innovation Way, Newark, 19711 Delaware USA; Department of Chemical Engineering, University of Delaware, Newark, Delaware USA

**Keywords:** *Clostridium acetobutylicum*, CoreReg, Regulation, Genome-scale metabolic model

## Abstract

**Background:**

Clostridia are anaerobic Gram-positive Firmicutes containing broad and flexible systems for substrate utilization, which have been used successfully to produce a range of industrial compounds. In particular, *Clostridium acetobutylicum* has been used to produce butanol on an industrial scale through acetone-butanol-ethanol (ABE) fermentation. A genome-scale metabolic (GSM) model is a powerful tool for understanding the metabolic capacities of an organism and developing metabolic engineering strategies for strain development. The integration of stress-related specific transcriptomics information with the GSM model provides opportunities for elucidating the focal points of regulation.

**Results:**

We describe here the construction and validation of a GSM model for *C. acetobutylicum* ATCC 824, *i*Cac802. *i*Cac802 spans 802 genes and includes 1,137 metabolites and 1,462 reactions, along with gene-protein-reaction associations. Both ^13^C-MFA and gene deletion data in the ABE fermentation pathway were used to test the predicted flux ranges allowed by the model. We also describe the CoreReg method, introduced in this paper, to integrate transcriptomic data and identify core sets of reactions that, when their flux was selectively restricted, reproduced flux and biomass-formation ranges seen under all regulatory constraints. CoreReg was used in response to butanol and butyrate stress to tighten bounds for 50 reactions within the *i*Cac802 model. These bounds affected the flux of tens of reactions in core metabolism. The model, incorporating the regulatory restrictions from CoreReg under chemical stress, exhibited an approximate 70% reduction in biomass yield for most stress conditions.

**Conclusions:**

The regulation placed on the model for the two stresses using CoreReg identified differences in the respective responses, including distinct core sets and the restriction of biomass production similar to experimental observations. Given the core sets predicted by the CoreReg method, remedial actions can be taken to counteract the effect of stress on metabolism. For less well-known systems, plausible regulatory loops can be suggested around the affected metabolic reactions, and the hypotheses can be tested experimentally.

**Electronic supplementary material:**

The online version of this article (doi:10.1186/s13068-014-0144-4) contains supplementary material, which is available to authorized users.

## Background

The organisms of the genus and class *Clostridium*, anaerobic Gram-positive Firmicutes, contain broad and flexible systems for substrate utilization [1]. Their inherent ability to use simple and complex carbohydrates, gases, and many other chemicals as substances to produce a wide range of products, such as carboxylic acids and various alcohols, underscores their unique potential as platform organisms for the production of chemicals and fuels [2]. In particular, *Clostridium acetobutylicum* has been the model organism for the production of butanol on an industrial scale through the acetone-butanol-ethanol (ABE) fermentation [1].

ABE fermentation is biphasic in nature; the acidogenic, exponential growth phase is characterized by the production of butyric and acetic acids, while the solventogenic stationary phase is characterized by the production of the ABE solvents. Production of acids and the resulting drop in the culture pH during the acidogenic phase drives the transition towards solventogenesis [1,2]. These metabolites, notably butyric acid and butanol, are toxic to the cells that produce them and affect their ability to function and eventually to survive. While several studies have been carried out to understand the changes during stress at various levels such as transcription [3-7] and translation [8], the impact of stress remains poorly understood at the systems levels in the context of the detailed cellular metabolism.

An important asset for understanding the metabolic capacity of an organism and deciding on metabolic engineering interventions is a genome-scale metabolic (GSM) model [9]. These models are network representations of the metabolic repertoire of an organism and are derived from genome-annotation information, metabolomic/fluxomic data, and biochemical characterizations. Advanced GSM models account for reaction stoichiometry and directionality, gene to protein to reaction (GPR) associations, reaction localization, transporter information, and biomass composition. They form a structured, multilayered framework for the integration and interpretation of experimental data and computational studies. These models computationally can direct engineering interventions in microbial strains for targeted overproduction of chemicals [10-13] and for elucidating the organizing principles of metabolism [14-17].

One of the earliest metabolic reconstructions was, in fact, a model of *C. acetobutylicum* [18]. A small stoichiometric model including core glycolytic, acidogenic, and solventogenic pathways was later generated [19]. These early models were used to examine how *C. acetobutylicum* produces butanol and byproducts such as acetate and butyrate. More recently, two GSM models of *C. acetobutylicum* ATCC 824 have been developed [20,21]. These models contain approximately 450 genes (that is*,* one-sixth of the number of genes coded on its genome). The Senger and Papoutsakis model [21] has recently been updated to include 242 additional reactions and contains a total of 490 genes along with thermodynamic constraints on the reversibility of reactions [22]. A larger, automatically generated model containing reactions associated with nearly 1,000 genes was constructed as part of the Model SEED effort [23]. However, all these models include only metabolic pathways without any information regarding metabolic changes in response to stressors. It is important to note that the activity and directionality of metabolic pathways under different conditions continue to be unraveled for *C. acetobutylicum*. The tricarboxylic acid cycle (TCA cycle), known to operate in a non-cyclic bifurcated manner, was recently shown to use *Re-*citrate synthase to produce α-ketoglutarate via citrate [24]. More recently, it has been shown based on ^13^C-metabolic flux analysis (^13^C-MFA) data that both α-ketoglutarate dehydrogenase (α-KGDH) and succinate dehydrogenase (SDH) are inactive during the acidogenic phase [25]. In contrast, the reaction that converts succinate to succinyl-CoA can carry flux in both directions [25]. While GSM models alone are quite useful for determining the metabolic potential of an organism, determination of the metabolic phenotype under various stress conditions requires the incorporation of additional information, such as transcriptomic data, which for now at least, are the most comprehensive, and genomically complete sets of genomic data that can be acquired.

A number of approaches have been proposed to incorporate regulatory information into GSM models. Regulatory flux balance analysis (rFBA) introduces Boolean constraints for gene expression into flux balance analysis (FBA) by linking the regulators to their targets in an iterative fashion [26,27]. The approach termed steady-state regulatory flux balance analysis (SR-FBA) combines the regulatory and metabolic models and solves the problem as a mixed-integer linear program [28]. GeneForce identifies incorrect regulatory rules and GPR associations in integrated metabolic and regulatory models [29]. PROM uses a probabilistic description of gene states and gene-transcription factor interactions while integrating heterogeneous high-throughput data [30]. The GIM3E method penalizes the flux for reactions whose associated genes have low expression levels in the transcriptome [31]. The recently proposed MTA method identifies minimal transformation rules from one metabolic state to another based on transcriptomic data [32] as in OptForce [33]. E-Flux modifies the maximum and minimum flux bounds of reactions as a function of the associated gene expression values [34]. All the aforementioned methods attempt to throttle back the flux in reactions associated with genes that are differentially expressed at a lower level. They differ in the use of penalty terms or bound contractions, threshold values for down-regulation, and the use of the parsimony criterion. CoreReg is fundamentally different, as it aims to explain the observed flux redirections as the consequence of a bound contraction of a small set of reactions (the core set). A hierarchy of core sets is identified (primary, secondary, tertiary, and so forth) by eliminating from consideration the dominant focal point of regulation and looking for additional modalities. This is analogous to the FORCE sets in the OptForce procedure [33]. For each one of the stress conditions we identify the minimal number of reaction fluxes (core set) whose change is sufficient to reproduce the flux ranges seen in the model when all regulatory constraints are imposed. The regulatory effect by the core set is propagated through stoichiometry throughout the model, recapitulating the experimentally observed changes. The method is described in detail in the Methods section.

In this paper, we describe the construction of a second generation genome-scale reconstruction of *C. acetobutylicum* ATCC 824, *i*Cac802, validation with experimental data. New reactions and pathways absent in earlier models include an updated TCA cycle, a completed fatty acid synthesis pathway, and additions to the purine, pyrimidine, and cobalamin biosynthetic pathways. The *i*Cac802 model along with the corresponding GPRs and metabolite information is available as SBML and excel files in Additional files 1 and 2 respectively. We also describe the use of the CoreReg method to integrate gene expression data into *i*Cac802 and predict nexus points of regulation, that underlie cellular response to the physiological stressors butanol and butyrate [3].

## Results

### Model comparisons

The GSM model *i*Cac802 for *C. acetobutylicum* ATCC 824 spans 802 genes and includes 1,137 metabolites participating in 1,462 reactions. All reactions present are elementally and charge balanced. GPR associations were determined from the available functional annotation information and homology predictions accounting for monofunctional proteins, multifunctional proteins, isozymes, and protein complexes. The model was curated to remove any thermodynamically infeasible cycles, as detailed in the Methods section. The *i*Cac802 model statistics and those of all other published models for *C. acetobutylicum* are shown in Table 1. *i*Cac802 has 64% more genes and 84% more reactions than the McAnulty *et al*. model [22]. *i*Cac802 contains a citrate synthase leading to a partial and bifurcated TCA cycle (based on the findings by Au *et al*. [25]), which is absent in the GSM by Lee *et al*. [20]. The latter model also does not predict the change from acidogenic phase to solventogenic phase under CO gassing conditions due to lack of internal protons [35] as reaction participants. This change is correctly predicted by *i*Cac802 as described in the model testing section. In addition, the GSM model by Lee *et al.* suggests that *Δadc* is lethal for cell growth due to coupling of succinate production with acetoacetyl-CoA production, contrary to experimental observations [36] and *i*Cac802 predictions. While all previous models contained an aggregate reaction for the production of hexadecanoyl-acp and hexadecanoyl-CoA from acetyl-acp and crotonyl-CoA, respectively, *i*Cac802 includes all participating reactions in fatty acid synthesis and metabolism pathways building up to these metabolites. *i*Cac802 also contains additional reactions from purine, pyrimidine metabolism, and cobalamin biosynthesis pathways (Additional file 3).

**Table 1:** **Genome-scale model comparison**

**Model statistics**	**Lee** ***et al*** **.** **[** 20]	**Senger** ***et al*** **.** **[** 21]	**McAnulty** ***et al*** **.** **[** 22]	***i*** **Cac802**
**Genes**	432	473	490	802
**Reactions**	502	522	794	1,462
**Metabolites**	479	422	707	1,137

The number of genes, reactions, and metabolites present in three previous genome-scale models of *C. acetobutylicum* and *i*Cac802.

### Model testing

**Figure 1:**
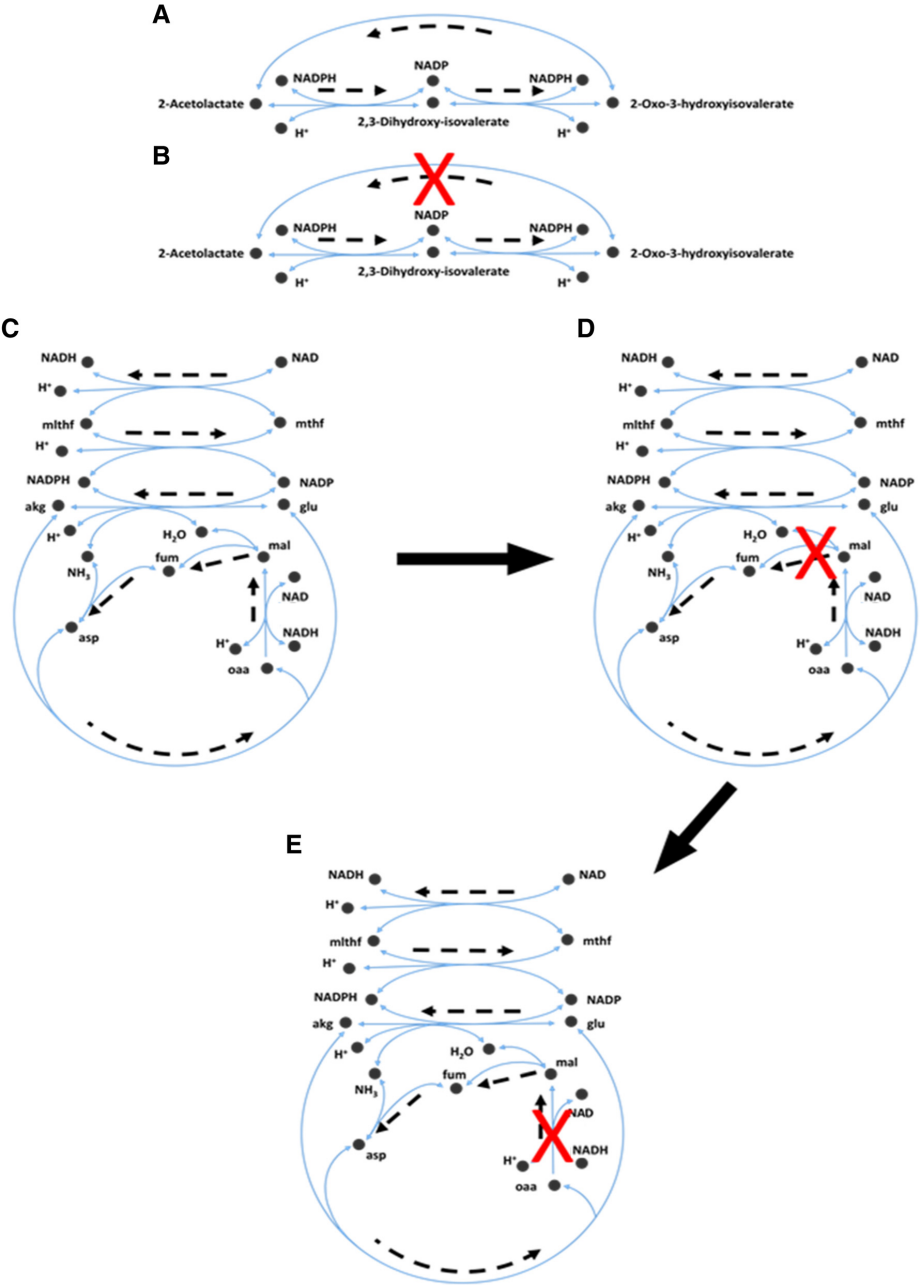
**Examples of thermodynamically infeasible cycles and their resolution.** Dashed lines indicate the direction of flux through the cycle forming reactions. **A)** A cycle containing three reactions. **B)** The fixed cycle from 1A after the removal of a single reaction with minimal literature evidence. **C)** A cycle containing seven reactions. **D)** The original cycle correction for 1C, involving the removal of a reaction with minimal literature evidence. **E)** The final cycle correction upon examination of ^13^C-MFA data [25].

The model was extensively tested to ascertain that it is capable of replicating flux ranges and phenotypes that have been documented for the wild-type (WT) organism and its mutants. The model predicted flux ranges were compared with experimental flux values from ^13^C-metabolic flux analysis (^13^C-MFA) [25]. The ^13^C-MFA data revealed that four reactions (pyruvate carboxylase (PC), fumarate hydratase (FH), pyridoxal phosphate synthase (PLPS), and alanine-glyoxylate (AGT)), which were originally removed to eliminate thermodynamically infeasible cycles, carried flux in the organism, and therefore, they were reinserted in the model. The cycles were instead eliminated by removing three alternate reactions (malate synthase (MS), succinate dehydrogenase (SDH), and malate dehydrogenase (MDH)) and by modifying the directionality of two others: succinyl-coenzyme A synthetase (SCS) was made reversible, and aspartate ammonia-lyase (ASPA) was restricted to the production of fumarate from aspartate. Figure 1C shows one of these cycles. ASPA was initially removed to fix the cycle due to a lack of literature evidence (Figure 1D), however subsequently MFA results indicated that this reaction carried flux whereas the MDH did not. Figure 1E shows how the addition of ASPA (directionally restricted) and removal of MDH avoids the formation of thermodynamically infeasible cycles while agreeing with experimental data.

**Figure 2:**
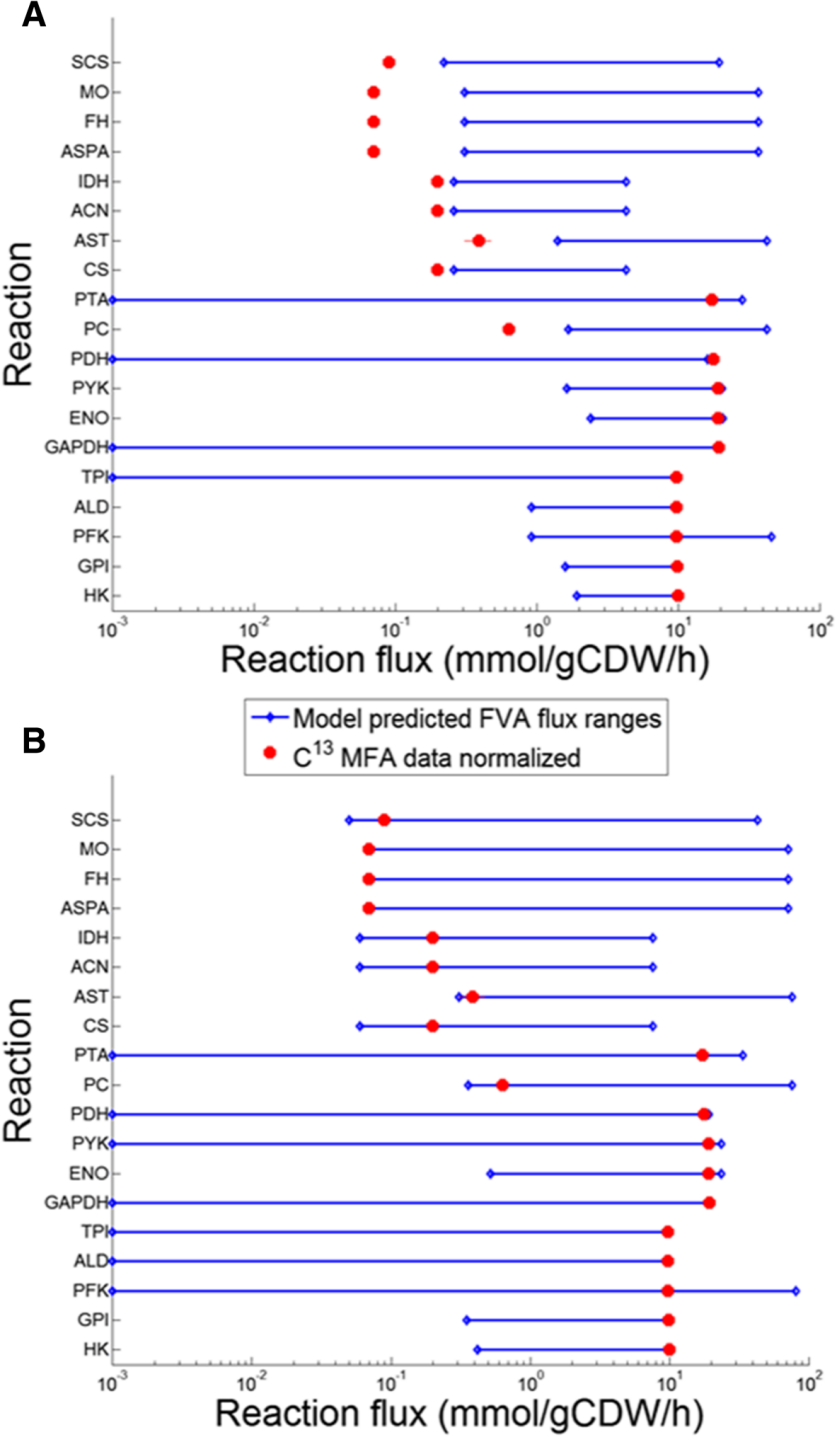
**Comparison of** ***in silico*** **and experimentally measured** ^**13**^ **C-MFA flux ranges for** ***C. acetobutylicum*** **[** 25]**. A)** under wild-type biomass constraint (0.32 mmol gDW^−1^ h^−1^, grown in complex media) [37]. **B)** under reduced biomass constraint (0.07 mmol gDW^−1^ h^−1^ , grown in defined media) given that the data were also collected during the transition to the solventogenic phase [25]. Hexokinase (HK) and pyruvate carboxylase (PC) had their experimental values outside the model predicted ranges under the reduced biomass constraint. FVA was performed with a glucose uptake rate of 10 mmol gDW^−1^ h^−1^. Full reaction names can be found in the list of abbreviations.

After these changes in the model, flux variability analysis (FVA) was performed on the core carbon metabolism reactions, and the flux ranges were compared to the values obtained by the ^13^C-MFA analysis [25]. These experiments were carried out with a chemically defined medium that results in slower growth and lower biomass yields. First, all fluxes were normalized for a glucose uptake of 10 mmol gDW^−1^ h^−1^. FVA was performed while constraining the growth rate to the WT value of 0.32 h^−1^ for *C. acetobutylicum* grown in complex media [37]. The comparison showed that the flux ranges of only four reactions (catalyzed by enolase (ENO), hexokinase (HK), pyruvate kinase (PYK), phosphotransacetylase (PTA), and phosphofructokinase (PFK)) encompassed the reported experimental values, as shown by Figure 2A. The reason for this is that *C. acetobutylicum* was grown in defined media by Au *et al*. [25], exhibiting significantly slower growth than in complex media [37]. In addition, the ^13^C-MFA data [25] was collected during the late growth phase with small amounts of solvents being produced, resulting in a reduced growth rate. Matching the FVA results with MFA data, we identified a growth rate value of 0.07 h^−1^. Upon reapplying FVA with the biomass yield constrained to 0.07 h^−1^ (see Figure 2B), all reactions except for HK and PC had flux ranges that encompassed experimental values. The two reaction experimental flux values differed from the model predicted range by only a value of 0.02 mmol gDW^−1^ h^−1^. It can be observed that, under these slow growing conditions, the TCA cycle reactions carry less flux and lie near the lowest end of the predicted flux range in Figure 2. The remaining flux is directed towards production of acids and solvents through pyruvate. This causes the flux of glycolytic reactions to lie near the high end of the predicted flux ranges (as shown by FVA predictions in Figure 2).

**Figure 3:**
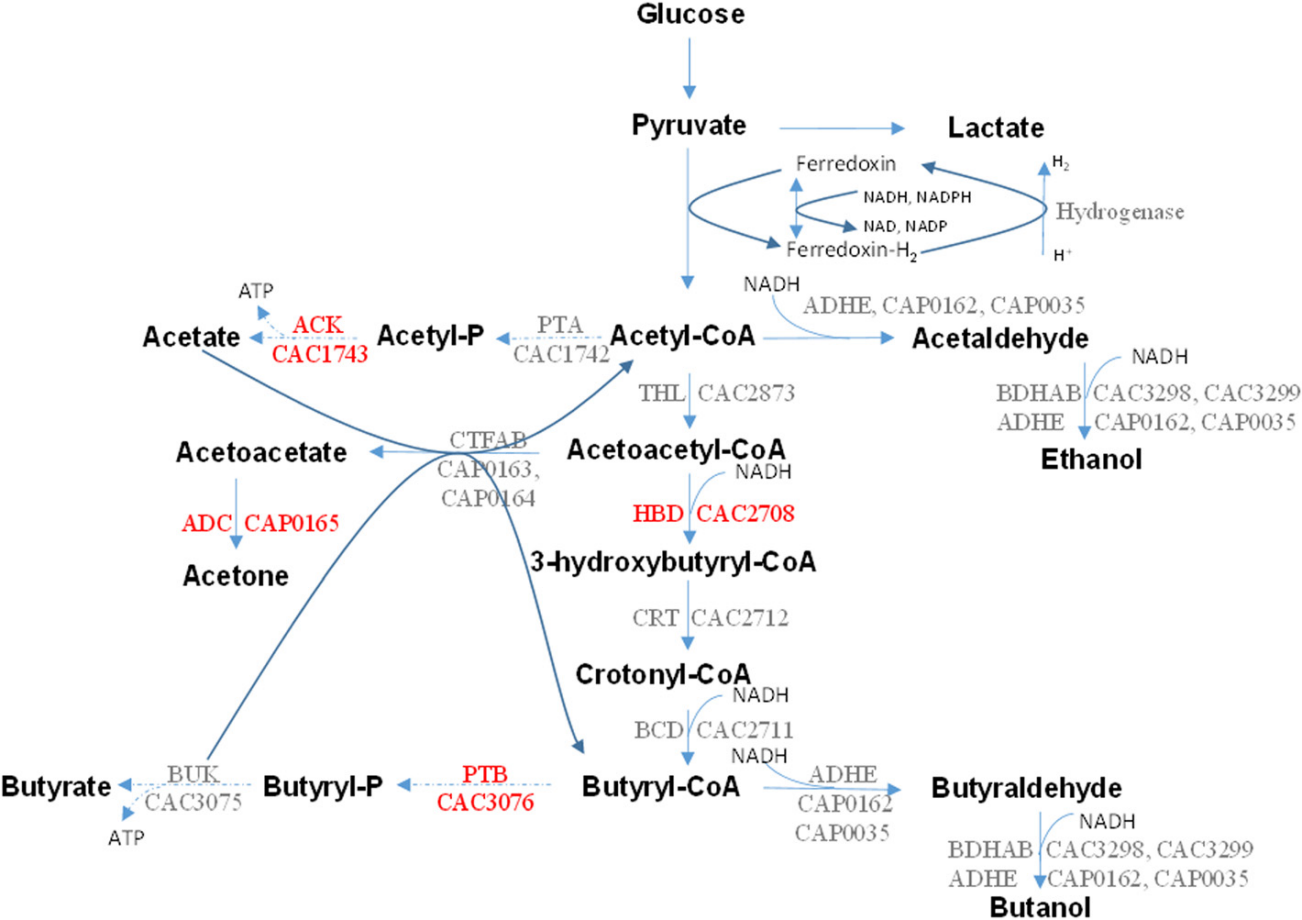
**The butyrate (butanoate) metabolism in** ***C. acetobutylicum*** **summarizing the formation of acids (acetic and butyric acid) and ABE solvents.** The acid formation pathways are represented by dotted lines. The mutants that were used to validate the GSM model are represented in red. (ACK - acetate kinase; PTA - phosphotransacetylase; ADHE - alcohol/aldehyde dehydrogenase; THL - thiolase; ADC - acetoacetate decarboxylase; CTFAB - CoA-transferase; HBD - hydroxybutyryl-CoA dehydrogenase; CRT - crotonase; BCD - butyryl-CoA dehydrogenase; BDHAB - butanol dehydrogenase; PTB - phosphotransbutyrylase; BUK - butyrate kinase).

Following the model updates and comparisons with ^13^C-MFA data, the model’s responses to gene knockouts and varying environmental conditions were also tested. The model was used to analyze the effect of increasing the size of the NADH pool on the production of various acids and solvents. It has been shown experimentally that an increase in the level of NADH leads to a concomitant increase in butyrate, solvents, and hydrogen production (Figure 3) [38]. Allowing for the free conversion between NAD and NADH resulted in an increase in their production with the exception of acetate, whose production was, as expected, found to be independent of reducing equivalent availability.

The model was also queried with respect to the ability to co-utilize glycerol. Glycerol as a highly reduced carbon source (its degree of reduction is 4.67 compared to 4.0 for glucose) allows for the generation of more reducing equivalents which drive the production of butyrate and alcohols (that is, butanol and ethanol). While *C. acetobutylicum* does not have the inherent ability to grow on glycerol as the sole carbon source, co-utilization of glycerol with glucose has been shown to result in a largely homo-butanol fermentation (that is, a fermentation where butanol is the predominant solvent produced) in *C. acetobutylicum* [39]. It is interesting to note that the glycerol uptake (CAC1319) and utilization (CAC1322) genes have been found to be up-regulated under butanol stress [3,5]. Based on this information, a glycerol uptake reaction was added to *i*Cac802 in order to test the impact of glycerol as a carbon source. The increased availability of reducing equivalents showed a similar affect, as having no redox constraint in the model by allowing for free interconversion between NAD + and NADH or NADP + and NADPH resulted in an increase in butyrate, solvents, and hydrogen production, as seen in Table 2.

**Table 2:** **Reducing equivalent dependence analysis of various acids, solvents, and hydrogen**

	**Glucose**		**Glycerol**
**Metabolites**	**With redox constraint**	**No redox constraint**	
Carbon source	10.00	10.00	20.00
Acetate	23.74	23.74	25.16
Butyrate	0.96	8.59	10.79
Ethanol	12.17	17.68	16.32
Acetone	7.34	17.01	17.04
Butanol	5.75	9.65	12.65
Hydrogen	31.18	35.85	61.73

Comparisons made between glucose with and without constraints on production of reducing equivalents and glycerol. Equivalent carbon flux values were chosen for both glucose and glycerol. All values except for carbon source uptake represent production fluxes with units of mmol gDW^−1^ h^−1^. Increasing availability of reducing equivalents led to increased product formation for all cases except acetate.

The model was further tested by showing that it can predict results from experiments examining the impact of CO gassing on product formation and cell growth [35]. CO gassing affects the cellular metabolism of *C. acetobutylicum* by forcing the transition from acidogenic to solventogenic fermentation (that is, initiating the uptake of butyrate and leading to the production of butanol and ethanol). CO inhibits the hydrogenase arresting H_2_ production (Figure 3) [35]. Therefore, the hydrogenase reaction flux was set equal to zero in the presence of CO. Since the organism has been shown to uptake butyrate during the CO sparging period [35], butyrate was supplied as an additional nutrient for the model. Using these additional constraints, the model predicted alcohol production (Table 3) during the acidogenic phase in accordance with experimental findings [35].

**Table 3:** **CO gassing analysis during acidogenic phase**

	**Wild type (0.52 h** ^**−1**^ **)**		**H inhibited (0.47 h** ^**−1**^ **)**	
**Metabolites**	**Lower flux bound**	**Upper flux bound**	**Lower flux bound**	**Upper flux bound**
Acetate	20.56	20.56	22.88	24.75
Butyrate	0.01	0.01	−1.99	−0.12
Acetone	0.00	0.00	0.00	0.00
Ethanol	0.00	0.00	0.00	1.87
Butanol	0.00	0.00	0.00	1.87
Hydrogen	13.02	13.02	0.00	0.00

Under CO gassing conditions the model shows inhibition of hydrogen production and butyrate uptake with alcohol but no acetone production. All the values represent production flux ranges with units of mmol gDW^−1^ h^−1^. A negative value indicates consumption instead of production. The numbers in parentheses indicate the maximum growth rate values.

Experimental data from fermentations using cell recycle were also examined using the GSM model. Cell-recycle conditions result in limited ammonia and phosphate uptake by the cells and an increase in overall alcohol production along with a reduction in biomass yield [40,41]. These conditions were simulated by restricting the flux bounds of ammonia and phosphate uptake reactions to an assumed 80% of their maximum allowable ranges determined by FVA [40,41]. The model showed a reduction in biomass yield and an increase in solvents yield, as shown in Table 4.

**Table 4:** **Cell recycling analysis during solventogenic phase showing lowering of biomass yield and increased solvent yield**

	**Wild type (0.32 h** ^**−1**^ **)**		**Cell recycle (0.17 h** ^**−1**^ **)**	
**Metabolites**	**Lower flux bound**	**Upper flux bound**	**Lower flux bound**	**Upper flux bound**
Ammonia	−2.94	−2.94	−2.35	−2.35
Phosphate	−0.37	−0.37	−0.30	−0.30
Ethanol	8.39	8.73	6.92	10.00
Butanol	0.00	0.52	0.00	4.04
Acetone	0.00	0.20	0.00	1.52

All the values represent production flux ranges with units of mmol gDW^−1^ h^−1^. Negative values indicate consumption instead of production. The numbers in parentheses indicate the maximum growth rate values. The solvent fluxes were converted to C mmol units to compare the overall solvent yields.

**Figure 4:**
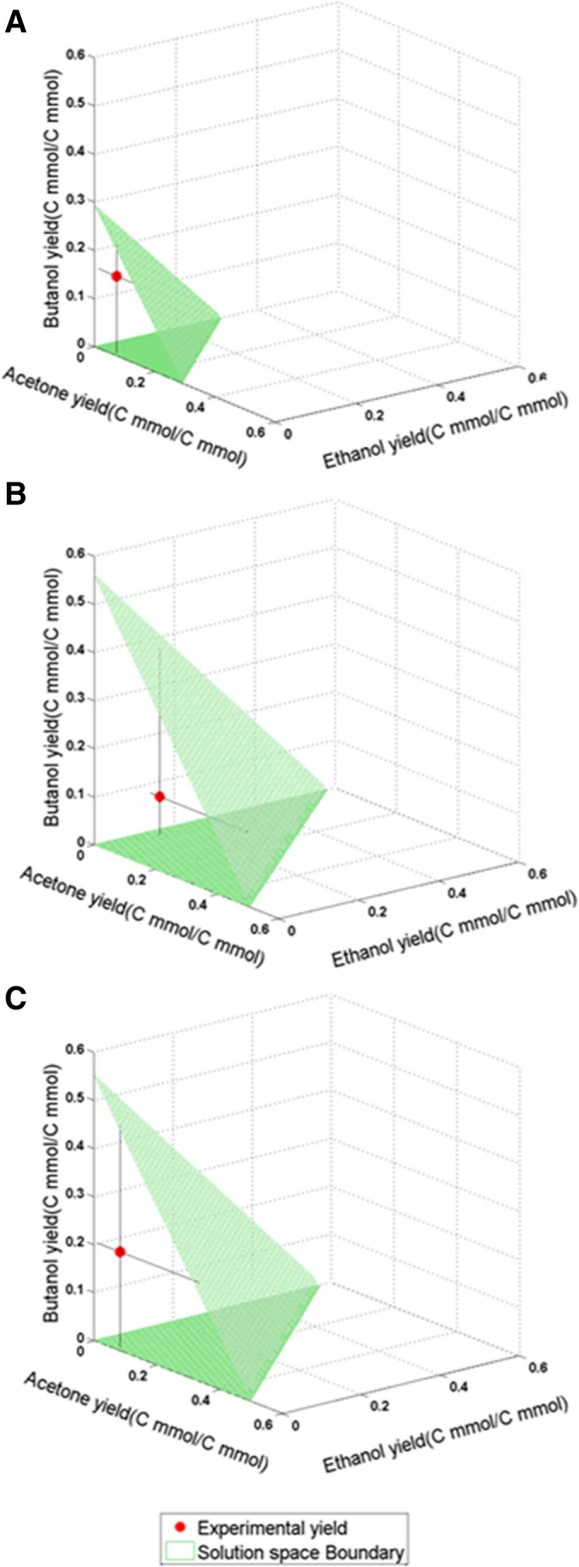
**Comparison of** ***in silico*** **and experimentally measured yields for solvents produced by** ***C. acetobutylicum*** **under the experimental growth rate constraints. (A)** Wild-type solution space with biomass constrained to 0.32 mmol gDW^−1^ h^−1^ [37]. **(B)** Δ*ptb* solution space with biomass constrained to 0.18 mmol gDW^−1^ h^−1^ [37]. **(C)** Δ*ack* solution space with max biomass constraint of 0.184 mmol gDW^−1^ h^−1^ [43].

**Figure 5:**
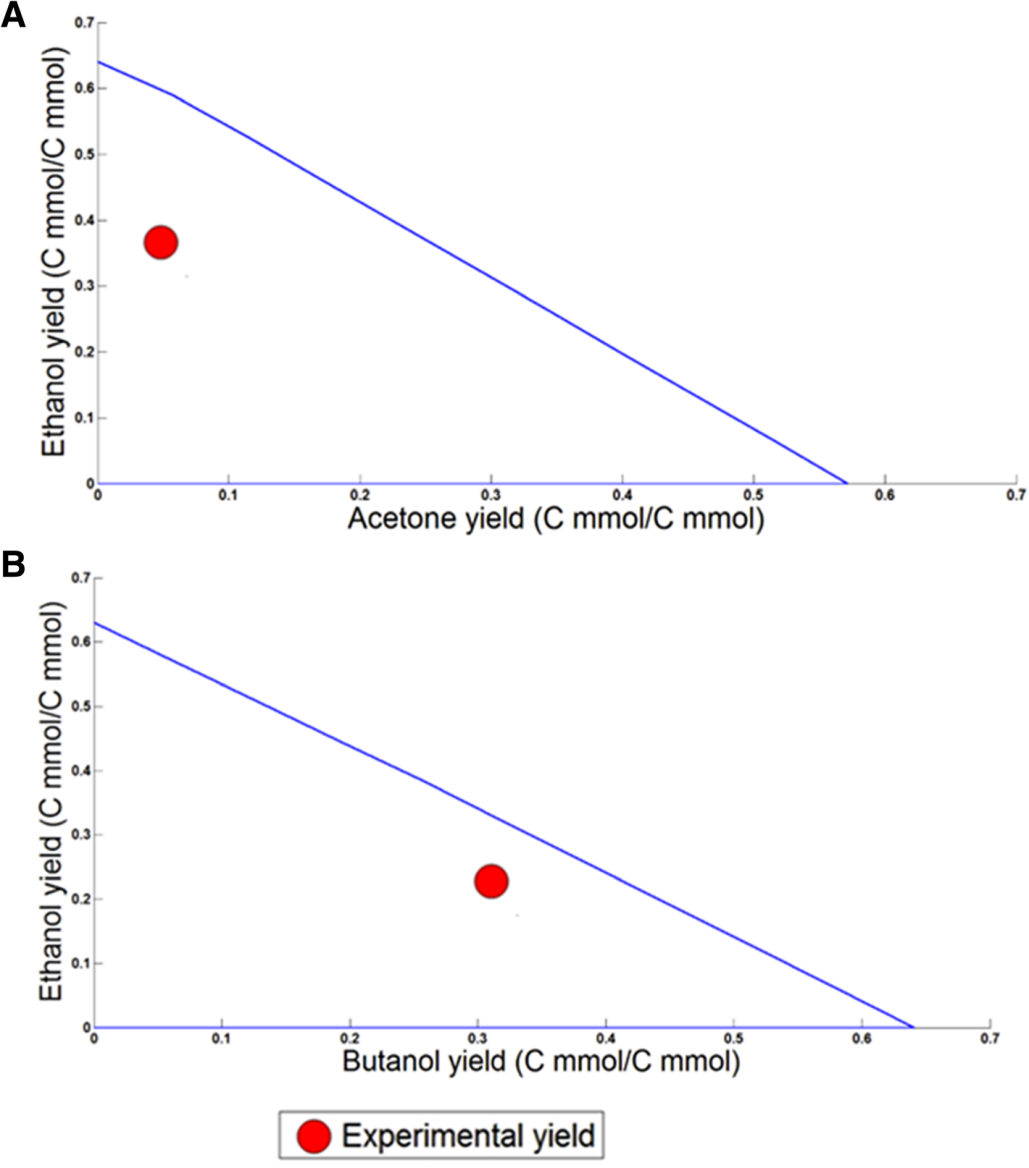
**Comparison of** ***in silico*** **and experimentally measured yields for solvents produced by** ***C. acetobutylicum*** **under the experimental growth rate constraint condition for the following strains.** **(A)** Δ*hbd* solution space for acetone versus ethanol yields with biomass constrained to 0.18 mmol gDW^−1^ h^−1^ [45], **(B)** Δ*adc* solution space for butanol versus ethanol yields with biomass constrained to 0.182 mmol gDW^−1^ h^−1^ [36].

Further model testing was performed by comparing experimental data of solvent yields for a number of *C. acetobutylicum* mutants [42] with *in silico* results. Mutants involving gene deletions affecting acid and solvent production in the ABE pathway were used to test *i*Cac802. Biomass was constrained to the reported growth rate values for the respective experiments. Reaction fluxes associated with a deleted gene were set to zero. FVA was performed to determine the possible range of solvent production. FVA was first performed with biomass constrained to the reported growth rate to evaluate the flux ranges for the produced acids and solvents. The identified flux ranges of solventogenic nutrients (glucose, acetate, butyrate, and carbon dioxide) were subsequently calculated by fixing both the growth rate and restricting the acids/solvents to the FVA calculated maximum and minimum values. Yield ranges were determined by evaluating the ratio of acids/solvents to the corresponding minimum nutrient flux. Mutants *∆ack* and *∆ptb* reduce (but do not eliminate) acetate and butyrate production by removing acetate kinase (ACK) and phosphotransbutyrylase (PTB) activities, respectively (Figure 3) [37,43]. For the two mutant strains, as well as for the WT strain, the model predicts a broad range of yields for the three solvents (butanol, acetone, and ethanol), as shown in the three-dimensional phenotypic solution space (Figure 4). This increased solvent production is also observed in experimental work by Jang *et al*. along with a reduction in acetate and butyrate production [44]. The study by Jang *et al.* also demonstrates that the butanol molar yield per glucose mole fed increases by 55% [44]. *i*Cac802 predicts that incorporation of these two knockouts results in an increase in butanol production by 63.6%. An earlier GSM model by Lee *et al.* [20] predicts an increase in butanol production but by a larger value of 86% due to a lack of internal protons in the model. In the case of mutant strains *∆adc* and *∆hbd*, acetone and butanol production is impaired [36,45] by knocking out acetoacetate decarboxylase (ADC) and hydroxybutyryl-CoA dehydrogenase (HBD), respectively (see Figure 5). In all cases the experimental yield is within the model-based calculated allowable yield for mutant phenotypes.

### Modeling metabolic stressors using the CoreReg

*i*Cac802 is a metabolic model and does not include any regulatory information. This section describes model regulation under various conditions by using transcriptomic data. Regulation was implemented in order to better describe the metabolism of *C. acetobutylicum* under butyrate and butanol stress and to pinpoint the reactions where flux changes are needed to explain the observed impact on biomass formation (that is, growth inhibition). Regulatory constraints on the *i*Cac802 model were imposed using the transcriptomic data from Wang *et al*. [3] in the form of modified reaction flux bounds for each of the stress conditions using the CoreReg method (see Methods section for full description).

**Figure 6:**
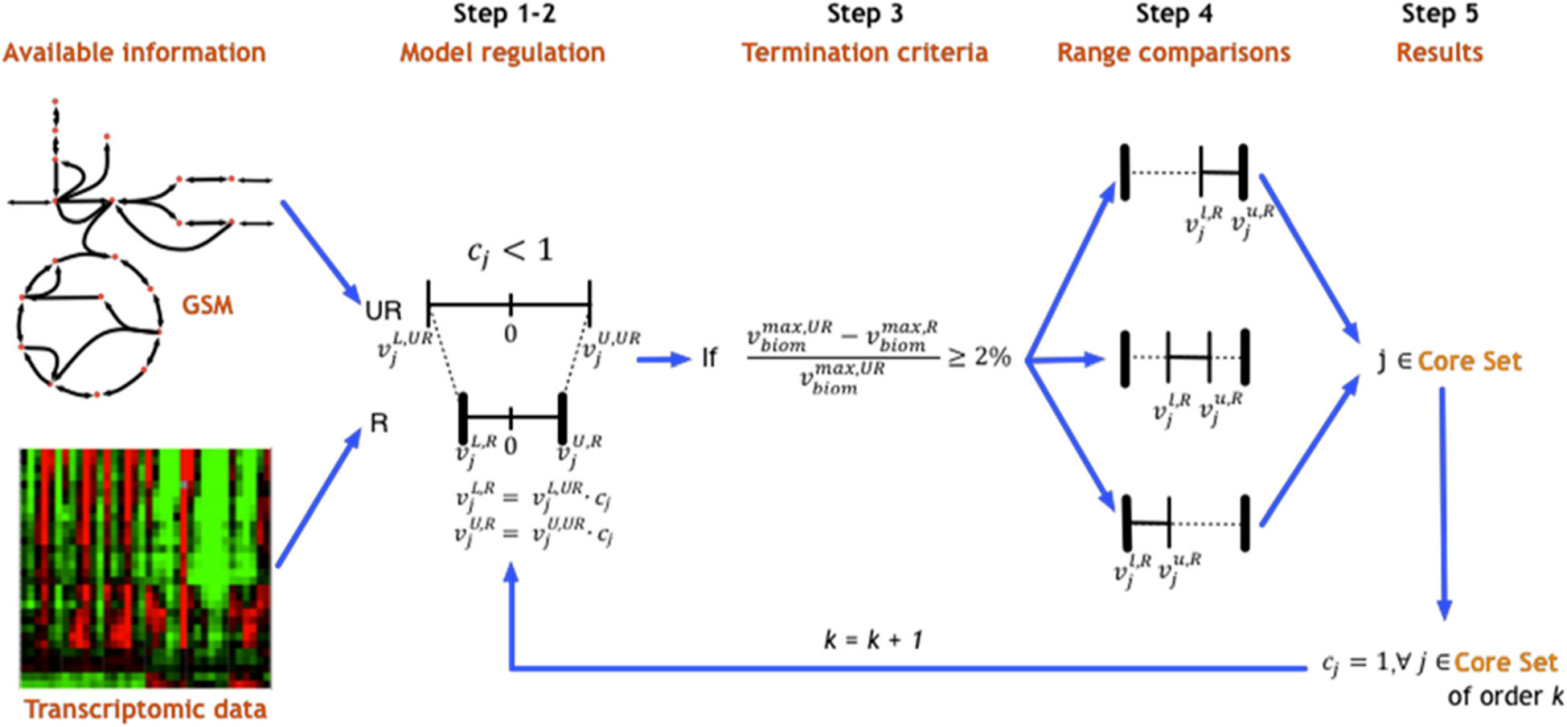
**Graphical representation of the CoreReg procedure.** The flux bounds for the unregulated model are identified (Step 1) and regulation is imposed on the model (Step 2) based on fold changes in the transcriptomic data. If the biomass regulation is greater than 2% (Step 3), then flux bounds from the regulated model are compared (Step 4) to their regulatory bounds to identify core sets of reactions. Regulatory effects of the identified core sets are excluded (Step 5), and the previous steps (Steps 2–5) are repeated to identify higher order core sets.

When regulation was imposed on the model, the biomass yield decreased by approximately 70% for all stress conditions except for the low-level butyrate stress, where the biomass yield was unaffected. For each one of the stress conditions we identify the reactions (core set) for which the application of regulatory constraints is sufficient to reproduce the flux ranges seen in the model when all regulatory constraints are imposed. Core sets of reactions were identified for each of the stress conditions by comparing flux bounds of the regulated model with the imposed regulatory constraints (Step 4 in Figure 6). The core sets represent likely nexus points of regulation under stress conditions, as they can broadly propagate the regulatory effect to the stress affected pathways through model stoichiometry. When regulatory bounds were imposed exclusively on the core set of reactions, the flux ranges of all reactions matched those of the model with all regulatory constraints. Subsequent core sets were found for the various stress levels by excluding the regulatory constraints on previously identified core sets (primary, secondary, tertiary sets, and so on). These subsequent core sets consisted of reactions whose regulatory constraints restrict the fluxes from wild-type distribution, and represent additional reactions that may be focal points of regulation. All these core sets are listed in Tables 5 and 6. In most cases, the same core sets of reactions were shared among the different levels of butanol stress. Three of the four reactions that made up these core sets (ornithine carbamoyltransferase (OCBT), arginosuccinate lyase (ARGSL), and arginosuccinate synthase (ARGSS)) belonged to arginine metabolism. OCBT was present in the primary core set of all levels of butanol stress. The final reaction, N-acetyl-gamma-glutamyl-phosphate reductase (AGPR), which is associated with amino acid metabolism, was present in the primary high-level butanol stress core set. The arginine metabolism genes identified in the core set for butanol stress are regulated by ArgR, the arginine repressor [3]. Expression of the genes corresponding to these identified arginine metabolic reactions was strongly down-regulated under butanol stress [3,5] with a corresponding effect on biomass formation (growth inhibition). Identification of reactions in the arginine metabolism using the regulated model and its corroborative evidence from transcriptomic studies confirms the key role of arginine metabolism in response to stress and its subsequent effect of growth and metabolism. Furthermore, apart from the arginine metabolism, these genes are also involved in the biosynthesis of proline and lysine, which further emphasizes their role in regulating the amino acid metabolism and hence growth and biomass formation.

**Table 5:** **Genes associated with core set of reactions under butanol stress conditions**

	**Stress level**	**ORF**	**Gene**
Primary (K=1)	Low	CAC0316	Ornithine carbamoyltransferase (OCBT)
	Med	CAC0316	Ornithine carbamoyltransferase
	High	CAC0316	Ornithine carbamoyltransferase
		CAC2390	N-acetyl-gamma-glutamyl-phosphate reductase (AGPR)
Secondary (K=2)	Low	CAC0974	Argininosuccinate lyase (ARGSL)
		CAC2390	N-acetyl-gamma-glutamyl-phosphate reductase
	Med	CAC0974	Argininosuccinate lyase
		CAC2390	N-acetyl-gamma-glutamyl-phosphate reductase
	High	CAC0974	Argininosuccinate lyase
		CAC2389	Acetylglutamate kinase (ACGK)
Tertiary (K=3)	Low	CAC0973	Argininosuccinate synthase (ARGSS)
		CAC2389	Acetylglutamate kinase
	Med	CAC0973	Argininosuccinate synthase
		CAC2389	Acetylglutamate kinase
	High	CAC0973	Argininosuccinate synthase
Quaternary (K=4) [minimal effect on biomass]	Low	CAC2391 or CAC3020	Bifunctional ornithine acetyltransferase/N-acetylglutamate synthase (OACT/AGS)
	Med	CAC2391 or CAC3020	Bifunctional ornithine acetyltransferase/N-acetylglutamate synthase
	High	CAC2388 or CAC0368	4-aminobutyrate aminotransferase acetylornithine/N-succinyldiaminopimelate aminotransferase (GABAT/ARGD)

If regulatory bounds were imposed exclusively on the reactions in the core set, the flux ranges would match those of the model with all regulatory constraints. Regulation was removed from the primary core sets to obtain secondary cores sets, and so on. Biomass regulation reduced with increasing order of core sets.

**Table 6:** **Genes associated with core set of reactions under butyrate stress conditions**

	**Stress level**	**ORF**	**Gene**
Primary (K=1)	Low [minimal effect on biomass]	CAC2113	Bifunctional pyrimidine regulatory protein PyrR/uracil phosphoribosyltransferase (UPRT)
	Med [minimal effect on biomass]	CAC0109 and CAC0110	Sulfate adenylyltransferase (SAT)
	High	CAC0973	Argininosuccinate synthase
Secondary (K=2)	Low		No core set
	Med		No core set
	High	CAC0316	Ornithine carbamoyltransferase
		CAC2389	Acetylglutamate kinase
Tertiary (K=3)	Low		No core set
	Med		No core set
	High	CAC0974	Argininosuccinate lyase
Quaternary (K=4)	Low		No core set
	Med		No core set
	High [minimal effect on biomass]	CAC2391 or CAC3020	4-aminobutyrate aminotransferase acetylornithine/N-succinyldiaminopimelate aminotransferase

If regulatory bounds were imposed exclusively on the reactions in the core set, the flux ranges would match those of the model with all regulatory constraints. Regulation was removed from the primary core sets to obtain secondary cores sets, and so on. Biomass regulation reduced with increasing order of core sets.

The primary medium level butyrate stress core set contained a reaction from pyrimidine metabolism, sulfate adenylyltransferase (SAT). The presence of this reaction can be related to the regulation of the DNA replication and repair mechanism which is initiated to protect the DNA from any damage owing to the oxidative stress component of the butyrate stress [3]. However, the subsequent core sets contained reactions involved in arginine metabolism, such as ARGSS in the high-level butyrate stress core set. Under butyrate stress, the effect that the regulatory constraints had on biomass yield was small. In comparison to butanol stress, under butyrate stress (low and medium), there is a strong up-regulation of genes in the arginine metabolism [3]. The addition of butyrate has a direct effect on the induction of solventogenesis, as the formation of solvents is directly related to reassimilation of butyrate from the medium (Figure 3). Jones *et al*. [46] have reported induction of the genes in arginine metabolism during the onset of solventogenesis, and this suggests that up-regulation of arginine genes under low and medium butyrate stress is associated with the induction of solventogenesis. The observation of arginine metabolism in the core set of high butyrate stress can be linked to the role of arginine metabolism as the acid response (AR3) system [3]. This ability of the model and regulatory modeling framework CoreReg to explicitly delineate the effect of two different metabolite stresses (at various levels) demonstrates the robustness and discriminatory capabilities of the model.

The addition of butyrate in the media leads to earlier onset of solventogenesis with higher butanol production [47,48], which is due to the corresponding up-regulation of the genes involved in solvent production and notably those of the *sol* operon (CAP0162-CAP0164, *adhe2*-*ctfA*-*ctfB*) [3,5]. The CoreReg method was able to simulate increased flux ranges for these reactions involved in solvent production during butyrate stress (Additional file 4).

## Discussion

A GSM model is a powerful tool that serves as a framework to visualize the changes captured from transcriptomic or proteomic data at the global metabolic level. The strength of such a model relies on the inherent characteristic capabilities to predict phenotypes from genotype. The proposed CoreReg method managed to elucidate focal points of regulation (core sets) on metabolic pathways. The core sets represent likely nexus points of regulation under stress conditions, as they can broadly propagate the regulatory effect to the stress affected pathways. Interestingly, different stressors and levels elicited different metabolic responses, as also corroborated by the DNA microarray data. The prediction of phenotypes and the corresponding regulation that leads to the phenotype along with model performance can be greatly enhanced by the development of a whole cell model. This would include the integration of regulatory knowledge derived from gene expression, transcription factors and their binding sites, regulation, and post-transcriptional regulation in the form of small non-coding regulatory RNA (sRNA) into GSM models. With the recent reconstruction of a transcriptional regulatory network [3] and the identification of the small RNome [4], the development of an integrated whole cell metabolic and regulatory model for *C. acetobutylicum* could provide superior insight into predicting phenotypes for the development of strains with higher tolerance to stressors and higher production of desired products. Thus, a more stress resilient strain of *C. acetobutylicum* may be engineered by improving these cellular functions.

## Conclusions

In this paper we have described the creation of a second-generation genome-scale metabolic model for *C. acetobutylicum* ATCC 824, *i*Cac802, and the use of transcriptomic data to apply additional constraints on reaction flux bounds using the CoreReg method. These constraints were calculated for varying levels of butyrate and butanol stress and were used to identify core sets of reactions whose changes in flux values can explain broadly all observed changes in metabolism.

CoreReg was able to differentiate between the two stressors, with a larger restriction on biomass for butanol stress. The core sets for butanol stress contain reactions in arginine and amino acid metabolism, while the butyrate stress core sets contain reactions in arginine and pyrimidine metabolism. These results corroborate previous findings concerning the down-regulation of arginine metabolism and regulation of DNA replication under stress conditions. Given transcriptomic data for other stressors or environmental conditions, the CoreReg method can be used to predict both the metabolic response and candidate focal points of regulation in terms of core sets. If there exists an available mechanistic description of the regulation, a remedial action can be taken to counteract the effect of stress on metabolism (for example, an up-regulating alternate pathway or a blocking regulator protein). In cases where the regulation mechanism is less well known, CoreReg results could be used to design plausible regulatory loops around the affected metabolic reactions. These regulatory hypotheses can then be tested experimentally.

## Methods

### Model construction

The general principles of the metabolic model reconstruction process have been previously described [49-51]. Construction of *i*Cac802 entailed the following processes: 1) identification of biotransformations using previous models and homology searches; 2) assembly of reaction sets into a genome-scale metabolic model and subsequent conversion into a computations-ready format; 3) identification and removal of thermodynamically infeasible cycles; and 4) evaluation and improvement of model performance when compared to *in vivo* growth phenotypes.

We began metabolic model reconstruction by parsing the existing genome-scale models for *C. acetobutylicum* ATCC 824 [20-22] and the automated model generated on the Model SEED website [23]. We also made use of MetRxn, a knowledgebase that includes standardized metabolite and reaction descriptions drawn from multiple databases and genome-scale models [52], to parse and compare the contents of these models. We converted the gene associations in the Model SEED to the same open reading frame (ORF) naming scheme as the other models and available experimental data (CACxxxx or CAPxxxx) using the start and stop sites and in the genome annotation at the Model SEED website and those in the TIGR gene annotation [53]. We examined and updated the elemental and charge balancing of all reactions by making use of the chemical formulas and charges provided by the SEED database (molecular charge values calculated at neutral pH) [23]. All reactions were checked to verify that the reactants and products shared the same total numbers of different atoms and the same total charge. Reactions that did not pass these tests were replaced with equivalent balanced reactions from Model SEED (Additional file 3). In the case of no alternate Model SEED reaction, the reactions underwent rebalancing, where protons, hydroxide, and/or water molecules were added to balance atoms and/or charges. Metabolites that were not fully specified due to the use of generic R groups as side chains, or that were oligomeric with a non-specified number of repeat units, were flagged for manual examination and balancing. One-to-one equivalency of metabolites with such generic R groups and metabolites without generic R groups was verified to ensure balanced reactions. The biomass equation was adapted from the McAnulty model [22], the only modifications being the use of charged and uncharged tRNAs to account for amino acid utilization, the doubling of the coefficients for solutes in the equation, and a slight decrease in the coefficient for protein use from 3.1 to 3.

Model curation began with the removal of thermodynamically infeasible cycles present in the model. Flux variability analysis (FVA) was performed with no biomass constraints to identify unbounded reaction fluxes. The number of unbounded reactions was reduced by restricting the directionality of certain reactions using Model SEED’s calculated values of the reaction’s Gibbs free energy. If the entire range, including error, was more than 4 kcal/mol removed from zero, the reaction was restricted to the direction specified by the free energy [54]. The method described by Schellenberger *et al*. [55] was then used to identify the core set of thermodynamically infeasible cycles which form the basis of all such possible cycles. This method relies on the observation that all possible thermodynamically infeasible cycles form the null space of the stoichiometric matrix [55]. During the identification of the basis of the null space, only the set of reactions whose fluxes hit the bounds during FVA were used to decrease processing time. The null space basis of this set was evaluated by LU decomposition of the stoichiometric matrix [55].

Of the cycles found, those involving only two reactions were examined first. These were composed of equivalent reactions, that is, reactions which both converted a given set of reactants to products or vice versa using the same co-factors, varying only in their directionality. One of the two identified equivalent reactions was subsequently removed based on a preponderance of literature or annotation information concerning its directionality. Cycles containing more than two reactions were formed due to the presence of pathways that recycled metabolites consumed or produced by an alternate route with a zero net metabolic cost. Reactions in these cycles were reviewed, and those with no literature or annotation reference were either removed or had their directionality restricted. Figure 1A is an example of such a cycle. The 2-acetolactate methylmutase (ACLM) reaction is more accurately represented by two separate reactions, (R)-2, 3-dihydroxy-3-methylbutanoate: NADP + oxidoreductase (R-DMBO) and 2, 3-dihydroxy-3-methylbutanoate: NADP + oxidoreductase (DMBO). The removal of this aggregate reaction eliminates the thermodynamically infeasible cycle, as shown in Figure 1B. Figure 1 (C-E) represents another cycle and its subsequent removal using experimental ^13^C-metabolic flux analysis (^13^C-MFA) data.

After reviewing all of the cycles in the null basis with up to five participating reactions, the null basis was reevaluated. After fixing the smaller cycles, the same review process was extended to larger cycles. This reevaluation process was iterated until no more cycles were detected using FVA. A total of 101 reactions were removed from the model, and 58 had their directionality restricted to stop the involvement of 404 reactions in thermodynamically infeasible cycles.

### Model simulations and analysis

Flux balance analysis (FBA) was performed to obtain the maximum attainable growth rate under the given constraints [56].

$$ \begin{array}{l} Max\kern0.5em {v}_{Biomass}\\ {} Subject\kern0.5em to\\ {}\begin{array}{ll}\kern1em {\displaystyle \sum_{j=1}^M{S}_{ij}{v}_j=0,\forall i\in 1,\dots, N}\hfill & (1)\hfill \\ {}\kern1em {v}_j^{\min}\le {v}_j\le {v}_j^{\max },\forall j\in 1,\dots, M\hfill & (2)\hfill \end{array}\end{array} $$

where *S*_*ij*_ is the stoichiometric coefficient for metabolite *i* in reaction *j. v*_*j*_ represents the flux of reaction *j*, while ${v}_j^{min}$ and ${v}_j^{max}$ denote the minimum and maximum flux bounds on reaction *j. N* and *M* represent the overall sets of metabolites and reactions, respectively.

FVA was used to identify reactions present in thermodynamically infeasible cycles.

$$ \left.\begin{array}{l} Max/Min\ {v}_{j*}\\ {} Subject\kern0.24em to\\ {}\begin{array}{ll}\kern1em {\displaystyle \sum_{j=1}^M{S}_{ij}{v}_j=0,\forall i\in 1,\dots, N}\hfill & (3)\hfill \\ {}\kern1em {v}_j^{\min}\le {v}_j\le {v}_j^{\max },\forall j\in 1,\dots, M\hfill & (4)\hfill \end{array}\end{array}\right\}\forall j*\in 1,\dots, M $$

No constraints were placed on the biomass in order to identify all thermodynamically infeasible cycles. This analysis was performed iteratively for all metabolites.

### Model testing

The model predictions were tested against experimental ^13^C flux data [25], experimental fermentation data [3,35,38,40,41], and *in vivo* gene knockout data from the literature [37,43,45,57].

While the acidogenic phase was simulated in the model solely through the inclusion of required nutrients, additional constraints were required for the solventogenic phase. The solventogenic phase of Clostridia is characterized by the uptake of acetate and butyrate along with the reduction of carbon flux towards amino acids [58]. Thus, the solventogenic phase was simulated with acetate and butyrate as additional nutrients, and constraints on the export of acids and excess amino acids.

### Incorporation of regulation using CoreReg

Regulation was incorporated into the model by a stepwise procedure that modified the minimum and maximum flux bounds of reactions based on fold change values of corresponding gene expression values by a new method called CoreReg. Gene expression data for each stress condition (GSE48031 and GSE48039), obtained from Wang *et al*. [3], were used to calculate the fold change from unstressed conditions using significance analysis of microarrays (SAM) [3,59]. Transcriptomic data were collected for three concentrations each of butyrate and butanol. We refer to these as low (30 mM butyrate, 30 mM butanol), medium (40 mM butyrate, 60 mM butanol), and high (50 mM butyrate, 90 mM butanol) stress conditions. The fold change for each reaction under each stress condition was calculated from gene expression fold changes under stressed conditions by using GPRs. In the case of multiple enzyme subunits, the minimum expression value for the genes associated with the subunits was considered for calculating the reaction fold change. In the case of isozymes the total transcript level, obtained by summation of all isozyme transcripts, was considered. The unregulated model reaction bounds without any biomass constraint represent the minimum and maximum possible bounds of each reaction. Thus, a further increase in these bounds does not affect any maximum yield calculations, as the bounds are not active. Thus, only down-regulated genes were considered for evaluating the regulated model. The procedure for implementing regulation can be divided into five steps as explained below (see Figure 6).

**Step 1**: FVA is performed on the unregulated (*UR*) model to obtain lower ${v}_j^{\mathrm{L},\mathrm{U}\mathrm{R}}$ and upper ${v}_j^{\mathrm{U},\mathrm{U}\mathrm{R}}$ reaction flux bounds. The value of k is set equal to one to indicate primary core set.

**Step 2**: FVA upper and lower bounds for the unregulated model are multiplied by the fold change value (*c*_*j*_) obtained using the transcriptomic data. The lower ${v}_j^{\mathrm{L},\mathrm{R}}$ and upper ${v}_j^{\mathrm{U},\mathrm{R}}$ bounds for the regulated (*R*) model are evaluated as follows:

$$ \forall j\ \mathrm{such}\ \mathrm{that}\ {\mathrm{c}}_j<1\to \left\{\begin{array}{c}\hfill {v}_j^{U,R}={v}_j^{U,UR}\cdot {c}_{j\kern1.25em }\kern5.5em (5)\hfill \\ {}\hfill {v}_j^{L,R}={v}_j^{L,UR}\cdot {c}_{j\kern1.75em }\kern5.5em (6)\hfill \end{array}\right. $$

Note that the updated lower bound is non-zero only for reversible reactions, effectively lowering the maximum possible flux value in the reverse direction.

**Step 3**: FBA is performed on the unregulated and the regulated model to obtain maximum biomass yields ${v}_{biom}^{\max, \mathrm{U}\mathrm{R}}$ and ${v}_{biom}^{\max, \mathrm{R}}$, respectively. If ${v}_{biom}^{\max, \mathrm{R}}$ varies from ${v}_{biom}^{\max, \mathrm{U}\mathrm{R}}$ by less than 2%, then the process is terminated, because the effect of the remaining regulation in the model is too small to cause any significant changes in metabolism as exemplified by the max biomass yield. Therefore, no additional regulatory core sets are extracted.

**Step 4**: FVA is performed at max biomass ${v}_{biom}^{\max, \mathrm{R}}$ on the regulated (*R*) model to obtain lower ${v}_j^{\mathrm{l},\mathrm{R}}$ and upper ${v}_j^{\mathrm{u},\mathrm{R}}$ reaction flux bounds. These bounds are next compared with the imposed regulatory constraints from Step 2.

**Step 5**: Reactions *j* whose flux bounds are equal to the regulatory constraints (that is, $$ {v}_j^{\mathrm{u},\mathrm{R}}\equiv $$$$ {v}_j^{\mathrm{U},\mathrm{R}} $$ or $$ {v}_j^{\mathrm{l},\mathrm{R}}\equiv $$$$ {v}_j^{\mathrm{L},\mathrm{R}} $$) are assembled into the core set of reactions (of order k). To identify secondary, tertiary, and higher order core sets, the fold change values (*c*_*j*_) for the previously determined k core sets are set to one, thus removing their regulatory role in the model. The process is repeated from Step 2 with the value of k increased by one.

## Additional files

Additional file 1:
**The genome-scale model** ***i*** **Cac802 with GPRs and metabolite information in SBML format.**
Additional file 2:
**The genome-scale model** ***i*** **Cac802 with GPRs and metabolite information.**
Additional file 3:
***i*** **Cac802 model comparisons with other** ***C. acetobutylicum*** **models.**
Additional file 4:
**Set of modified reaction bounds and analysis for all six stress conditions.**

## Abbreviations

^13^C-MFA: ^13^C-metabolic flux analysis
AADC: acetoacetate decarboxylase
ABE: acetone-butanol-ethanol
ACGK: acetylglutamate kinase
ACK: acetate kinase
ACLM: 2-acetolactate methylmutase
ACN: aconitase
AGPR: N-acetyl-gamma-glutamyl-phosphate reductase
AGS: N-acetylglutamate synthase
AGT: alanine-glyoxylate
ALD: aldolase
ARGD: N-succinyldiaminopimelate aminotransferase
ARGSL: arginosuccinate lyase
ARGSS: arginosuccinate synthase
ASPA: aspartate ammonia-lyase
AST: aspartate transaminase
CS: citrate synthase
DMBO: 2,3-dihydroxy-3-methylbutanoate:NADP + oxidoreductase
ENO: enolase
FBA: flux balance analysis
FH: fumarate hydratase
FVA: flux variability analysis
GABAT: 4-aminobutyrate aminotransferase acetylornithine
GAPDH: glyceraldehyde 3-phosphate dehydrogenase
GPI: glucose-6-phosphate isomerase
GPR: gene-to-protein-to-reaction
GSM: genome-scale metabolic (model)
HBDH: hydroxybutyryl-CoA dehydrogenase
HK: hexokinase
IDH: isocitrate dehydrogenase
α-KGDH: α-ketoglutarate dehydrogenase
MDH: malate dehydrogenase
MS: malate synthase
OACT: ornithine acetyltransferase
OCBT: ornithine carbamoyltransferase
PC: pyruvate carboxylase
PDH: pyruvate dehydrogenase
PFK: phosphofructokinase
PLPS: pyridoxal phosphate synthase
PTA: phosphotransacetylase
PTB: Phosphotransbutyrylase
PYK: pyruvate kinase
R-DMBO: (R)-2,3-dihydroxy-3-methylbutanoate:NADP + oxidoreductase
rFBA: regulatory flux balance analysis
SAM: significance analysis of microarrays
SAT: sulfate adenyltransferase
SCS: succinyl coenzyme A synthetase
SDH: succinate dehydrogenase
SR-FBA: steady-state regulatory flux balance analysis
sRNAs: small non-coding regulatory RNAs
TPI: triosephosphate isomerase
UPRT: uracil phosphoribosyltransferase
